# May Thurner syndrome revealed by left calf venous claudication during running, a case report

**DOI:** 10.1186/s13102-018-0092-6

**Published:** 2018-02-02

**Authors:** Samuel Béliard, Damien Feuvrier, Emilie Ducroux, Lucie Salomon du Mont

**Affiliations:** 1 PEPITE EA4267, Platform Exercise Performance Health Innovation (EPHI), University Bourgogne Franche-Comté, F-25000 Besançon, France; 2 Cardiology, Angiology, Anticoagulation Clinic, Hôpital Louis Pasteur, Dole, 39100 France; 3 Department of orthopedic and trauma surgery, plastic surgery, plastic and reconstructive, hand surgery, University Hospital , CHUR Jean Minjoz, 25000 Besançon, France; 4 Laboratory of Anatomy, Faculty of Medical and Pharmaceutical Sciences, 20 rue Ambroise Paré, 25030 Besançon, France; 5 Vascular Surgery, Vascular Medicine, University Hospital, CHUR Jean Minjoz, 25000 Besançon, France; 6 EA. 3920, Unversity of Franche-Comté, Besançon, France

**Keywords:** May Thurner syndrome, Venous compression, Venous claudication, Post thrombotic syndrome, Case report

## Abstract

**Background:**

May Thurner syndrome is relatively unknown to physicians, its management is well standardized and the outcomes of treatment are satisfactory in the short to medium term.

**Case presentation:**

We report the case of a patient who suffered from venous claudication during running which impaired their quality of life, decreased their athletic performance and resulted in a career change. May Thurner syndrome diagnosis was made after extensive hemodynamic analysis of a lower limb venous duplex ultrasound scan. This diagnosis was later confirmed by imaging. Subsequent endovascular care provided rapid and sustained clinical improvement.

**Conclusion:**

The main difficulties with the May Thurner syndrome are to think about it and know how to look for it; indeed the misdiagnosis time can be long. When diagnosis is made, treatment could be easy and effective.

## Background

The most commonly proposed aetiologies of calf pain during physical exercise are muscular (muscle injury, compartment syndrome), rheumatologic (chronic bone micro traumatic pathology, projected joint pain), neurological (lumbar compression syndrome, neuropathy device) or arterial (claudication). Chronic venous disease that is related or not with a post thrombotic syndrome, could be another explanation but this is not often explored and therefore not often suggested as an aetiology. Symptoms of venous claudication during physical exercise involve deep pain, first dull, then intense and constrictive, in the calf or thigh, culminating in functional impairment. Unlike arterial claudication, these sensations don’t diminish after effort, but dissipate when the patient assumes a position that enhances venous drainage position [1]. Often this involves elevating the lower limbs to 45 ° relative to the horizontal when in the supine position [2].

Here we report a clinical case in which investigative semiology analysis and hemodynamic exploration for venous claudication after exercise helped to diagnose May Thurner syndrome (MTS) in a patient, following a lengthy period of misdiagnosis. We obtained the written agreement of the subject involved in the clinical case before writing this article. Then the subject signed the declaration for the ‘Consent for publication’.

## Case presentation

### Context

A 25-year-old man was referred to vascular medicine clinic because of pain in the left calf while running, which had been occurring for the past 4 years.

This patient practiced almost daily physical activity that involved both combative and running (over 8 h per week), and he competed at a regional level (with a personal best of running of 36min12s/10000 m). In 2012, the patient had reported left calf pain when running, forcing him to stop. He described a calf swelling sensation that gradually increased with the intensity of running. While in the past he could easily run 15–20 km, the patient reported at that date that he could not run more than 2 km before having to stop. After stopping, the pain was deep and persisted for several minutes; it was also associated with swelling of the calf. This pain had led him to change his occupation to a less physically demanding job (military officer in the past, and now security officer). The consequences were a weight gain of 8 kg, and a cessation of all physical activity (running, fight sports). The patient also reported a loss of self-esteem.

### History of the disease

Over a period of 4 years, the patient was seen by two general physicians, a rheumatologist, a neurologist, a sports physician and a vascular physician. Many explorations were carried out (spinal and pelvic x-ray, pelvic and lumbar spine scan, venous duplex scan, arterial duplex scan, and electromyography), all of which revealed no sign of dysfunction. The sports physician raised the possibility of diagnosis of a compartment syndrome, but the patient refused to submit to investigation of intramuscular pressure taken at rest and in immediately post exercise. Different therapies were proposed, including anti-inflammatory drugs, paracetamol and compression stockings (Class 2).

The patient interview revealed that a proximal deep vein thrombosis DVT (thrombosis in the iliac, femoral and popliteal veins) of the left lower limb had been diagnosed in 2012 and treated for 6 months by direct oral anticoagulant therapy (Rivaroxaban, Xarelto 20 mg per day) combined with compression stockings french class 3 worn (20–36 mmHg) for 6 months and then class 2 compression socks (15–20 mmHg) thereafter. This episode of DVT occurred spontaneously without major or minor contributing factors. There was no family history of venous thromboembolism disease. Clinical and laboratory tests had found no neoplasia. The biological thrombophilia test at distance of anticoagulation was negative.

### First consultation

#### Physical examination

The patient was 1.78 m and 79 kg (body mass index = 24.9 kg/m^2^). He was afebrile, and his brachial blood pressure was 120/70 mmHg. Upon examination the legs were hot and there was no trophic disorder. There was a difference in the circumference of the calves (left calf 2 cm > right calf). There were no visible or palpable varicose veins. Peripheral pulses were present and there was no sign of Lassegue. Palpation of the major lower limb joints (ankles, knees, hips) was not painful. Calves were flexible, painless, with good trophicity. No lymphadenopathy was found and no local inflammatory signs. When examined by auscultation, there were no vascular sounds along the vascular axes.

#### Vascular duplex scan exploration

All Doppler ultrasound measurements were performed with a high-resolution ultrasound machine (Affiniti 70, Philips, Amsterdam, The Netherlands). The deep venous system was free and compressible when compressed by the ultrasound probe at calf level (posterior tibial veins, peroneal, gastrocnemius and soleus), at femoral popliteal level (popliteal veins, femoral and common femoral) and at ilio-cava level with no sign of recent deep vein thrombosis. There was wall thickening at the left common femoral vein CFV (anterior posterior diameter APD under compression = 3.9 mm) and at the left external iliac vein (APD under compression = 3.6 mm); this finding had already been highlighted when vascular physician examined the patient in 2013.

The Doppler examination showed a clear asymmetry of the respiratory modulations of blood velocity between right and left CFV. In the left CVT, respiratory modulations were almost absent and blood volume flow was not as high as on the right side. We found a reflux into the left internal iliac vein and dilation of the left gonadal vein. While standing, there was no significant deep venous reflux (> 1 s) at the popliteal vein (functional venous valves). The superficial venous system (great and small saphenous veins) was compressible and continent.

The diagnostic hypothesis of MTS was issued at the end of this first consultation, taking into account the following clinical and hemodynamic arguments:

- ▪ History of the left proximal DVT without predisposing factor.
- ▪ Symptoms of left calf venous claudication due to effort (+/− associated with unilateral edema reported by the patient).
- ▪ Asymmetry respiratory modulation and venous blood volume flow at the left proximal deep vein (as a result of a downstream obstacle).

Additional morphological exploration was requested computed tomography angiography (CTA) and a surgical opinion was recommended and requested.

### Support

#### CTA acquisition with venous phase

**Fig. 1:**
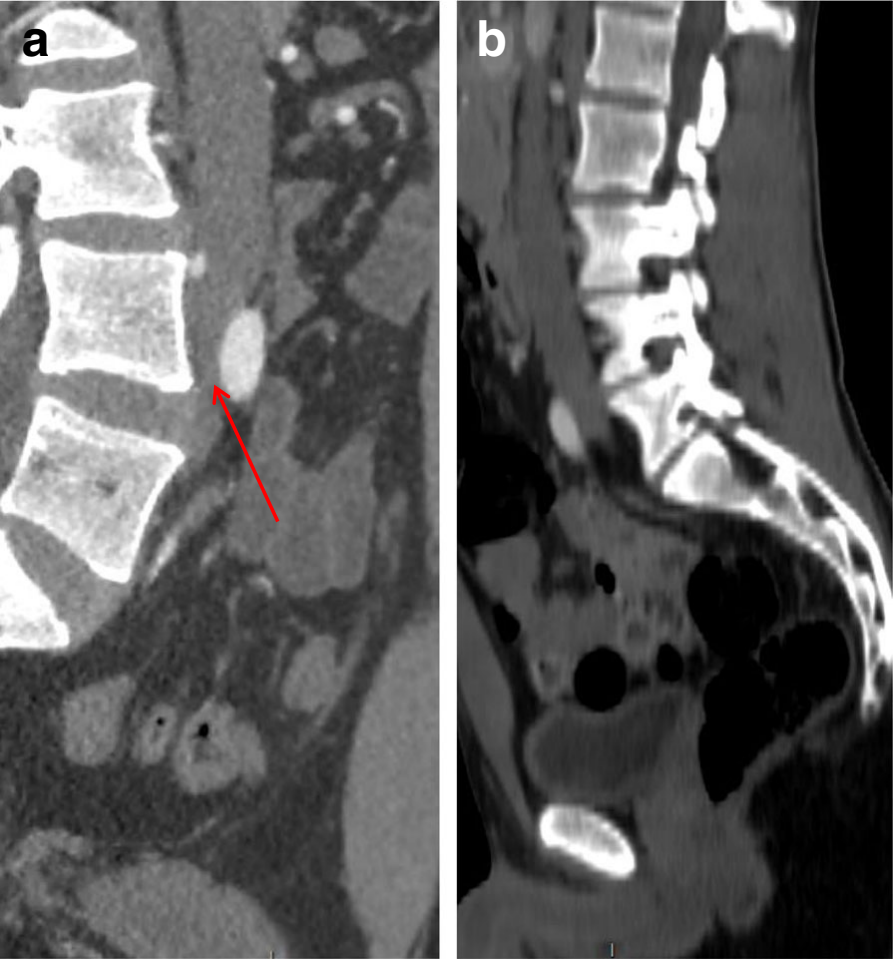
computed tomography angiography. **a**. Compression of the left common iliac vein between the right common iliac artery and lumbar spine. **b**. Normal left common iliac vein

The CTA revealed compression of the left common iliac vein (CIV) between the right common iliac artery (CIA) and lumbar spine (Fig. 1) and intraluminal spurs. There was also dissimilar venous systems collatorally with that was more highly developed on the left iliac axis relative to the right iliac axis, reflecting the need to create a system of substitution.

#### Surgical procedure

**Fig. 2:**
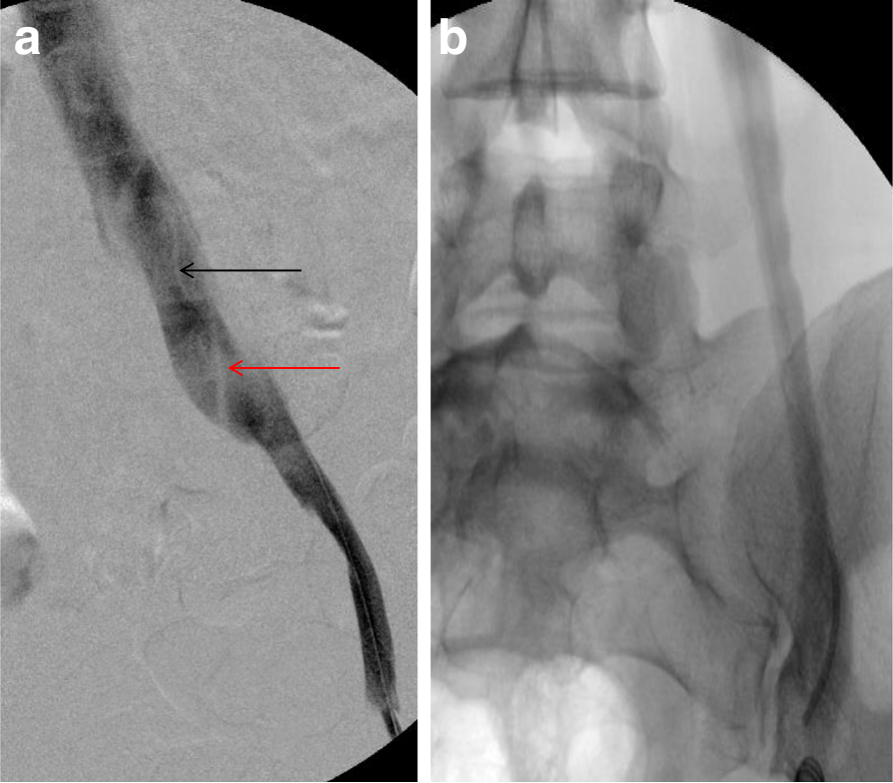
initial venography, **a**. MTS. →Synechias endoluminal of the left common iliac vein. **b**. Normal left common iliac vein

In this context of symptomatic MTS with compatible imaging, venography was performed in order to confirm the diagnosis and to treat the MTS at the same time. Venography showed patency of the left venous axis iliac featuring endoluminal spurs and a footprint at the terminal portion of the iliac vein (Fig. 2).

Endovascular support was carried out with introduction of two Wallstent endo prostheses (Boston Scientific) into the common iliac and external iliac veins, associated with angioplasty intra stent to modulate the different areas of overlap, the diameters and lengths to achieve a harmonious assembly. The phlebographic control carried out at the end of the procedure was satisfactory.

### Results at 3 and 6 months

At 3 and 6 months, the patient reported an improvement in symptoms. The patient restarted running, and initially venous claudication was not felt and the patient was once more able to run more than 10 km. He currently wears a compression class III especially during physical activity, as advised.

Angiographic assessment found normal stent patency at the iliac vein, with symmetrical respiratory modulations. It was also decided to carry out a duplex ultrasound scan at 3 months. At this scanning session the stents were in place and permeable. Pharmacological treatment in the form of an anti platelet (Clopidogrel 75 mg) was prescribed.

## Discussion

With this study, the diagnostic criteria of MTS by imaging (CTA and venography) and the subsequent therapeutic management are now well described and outcomes suggest excellent recovery.

The difficulty in diagnosis and treatment of MTS is simply to consider it a possibility before overt clinical presentation and then to carry out the appropriate explorations. For this, the vascular physician should be primarily a clinician and should strive to conduct the investigation via a directed examination and a search for non-specific clinical signs. Then during functional exploration, the physician must consider the hemodynamics and not base their assessment on imaging alone. Indeed, the compression of the iliac vein is not detectable by duplex scan and in the absence of acute proximal deep vein thrombosis (external iliac or at the femoral vein), the physician can miss a diagnosis of MTS. Respiratory modulations are spontaneous rhythmic flow which reflect the effects of pulmonary ventilation on the variability of venous flow. During inspiration, diaphragmatic contraction increases the abdominal pressure, and reduces the flow of the lower limb veins that are dependent on the inferior vena cava system. The opposite phenomenon is observed during expiration [3]. If a hemodynamic exploration includes analysis of respiratory modulations of the proximal venous flow (within the external iliac vein and / or the femoral vein), accompanied by dynamic maneuvers (Valsalva, venous blood volume flow), the presence of asymmetry between the two sides would point quite clearly to a downstream stenosis syndrome (the obliteration table, or compression table is to be explored in static imaging thereafter).

In clinical practice, diagnosis and treatment of MTS presents several difficulties.

- ▪ Physicians do not clearly understand the aetiological in its entirety. The misdiagnosis time can be long, a study of 58 patients with MTS revealed a median time of 59 +/− 15 months (range: 0–376 months) before the diagnosis [4]. The clinical case reported here confirms this problem, with 4 years before obtaining suitable support.
- ▪ In the case of clinical suspicion, the difficulty is to establish the diagnosis. It is important to differentiate a “Cockett footprint “(compression of the vein by the artery on a static image without endoluminal spurs) or “Cockett postural syndrome “(compression-related hyperlordosis) with specific MTS which associating compression of the left iliac vein by the right iliac artery, and the presence of endoluminal spurs within the iliac vein [5]. A high frequency of this anatomical feature is found in many asymptomatic patients both on autopsy analysis (22% of 430 bodies) [6] and/or imaging [7]. Kibbe et al. [7] demonstrate, on a CTA series of 50 consecutive patients evaluated for emergency abdominal pain, without any venous symptoms, a reduction > 50% in diameter of the left CIV in a quarter of the patients, and > 25% in two thirds of patients. Similarly, a study from Narayan et al. [8] shows that a compression < 70% does not increase the relative risk of DVT [8], while a compression > 70% of the vein increases the risk. The presence of a Cockett footprint on CTA [8] or angiography combined with magnetic resonance imaging [9] of the iliac vein to the iliac artery is not sufficient to confirm the diagnosis.

**Fig. 3:**
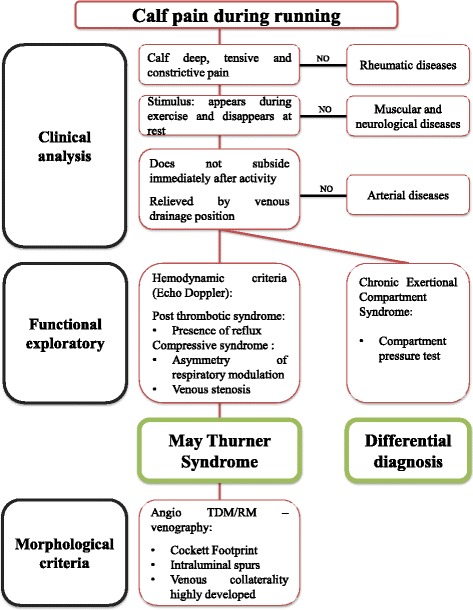
Decision tree: reasoning before an exertional leg pain

The differential diagnostic process before a calf venous claudication, should be structured to consider a diagnosis of MTS (Fig. 3). Diagnosis of MTS should thus be established based upon a set of arguments [10]:

- ▪ Medical history: A history of venous thromboembolism, acute symptoms (left proximal DVT), chronic symptoms (post thrombotic syndrome PTS, recurrent DVT, venous claudication, unilateral varicocele [11]). PTS is defined by the presence of symptoms (pain, heaviness, pruritus) and/or chronic venous signs (C1 – C6 of CEAP (Clinical, Etiologic, Anatomic, Pathophysiologic) classification: telangiectasia, reticular veins, varicose veins, edema pigmentation, eczema, lipodermatosclerosis, white atrophy, venous ulcer) secondary to lower limb deep venous thrombosis [12].
- ▪ Clinical: With MTS, pain is mainly located in the calf. This pain is deep and constrictive. It appears during exercise and disappears at rest. Unlike arterial claudication, MTS pain does not yield immediately after activity and is relieved by assuming a venous drainage position. All characteristics of this pain often mirror those of chronic exertional compartment syndrome [13]. To differentiate both entities, a compartment pressure test must be carried out. Sometimes, more than one aetiology for exertional leg pain can coexist in an athlete [14].
- ▪ Hemodynamic: Duplex ultrasound scan [15] (PTS with analysis of the reflux, compressive syndrome with asymmetry respiratory modulations, venous stenosis), +/− plethysmography (evaluation of reflux) +/− intravenous ultrasound [16] (intra luminal damaged, pretreatment evaluation) needs to be performed and must involve specific evaluation of the respiratory modulation of venous flow bilaterally.
- ▪ Morphological [17] (CTA +/− angio MR +/− venography): Evidence of compression, more highly developed collatorally, the presence or absence of thrombosis and intra luminal spurs.

If MTS is confirmed, therapeutic management is now well defined [18], and is relatively simple and minimally invasive. It combines medical treatment [4] (walking, compression device class III) and endovascular management [19] (angioplasty stenting). This support leads to a significant improvement in symptoms and quality of life [20], and is associated with high low long-term patency rates [4, 10]. The main risk of this therapeutic management is recurrence. However, medium- and long-term patency have been evaluated and are highly in favor of treatment [4] with primary patency rates of 74.1% at 1 year (SE, 6.3%) and 38.1% at 60 months (SE, 12.4%); and secondary patency rates of 85.8% at 1 year (SE, 5.0%) and 73.8% at 60 months (SE, 9.7%). There is no consensus concerning the duration of platelet therapy after the surgical procedure. In our practice, we continue this treatment after the first year by re-evaluating the risk benefit ratio.

## Conclusion

In conclusion, diagnostic error before a MTS, reported in the literature and found in this clinical case could be reduced by changing two factors. First a better understanding of this disease and of venous claudication indices by physicians is required (general, vascular and sports medicine physicians). Second a systematic hemodynamic analysis by the vascular physician of the venous vasculature lower limbs is needed. Compliance with this latter element will highlight asymmetry of venous flow to the proximal lower limb. A report of the French Society for Vascular Medicine [21] recommends an exploration of the iliac cave veins with analysis of respiratory modulation and examination of venous blood volume flow. Laroche [22] recalled the importance of this comprehensive review of the venous system at the 50th congress of the French college of vascular disease [22].

### Practical implications

- The May Thurner Syndrome is not well known, which can cause a long delay between the onset of symptoms and treatment.
- The diagnostic criteria, before a calf venous claudication, should be structured in order to reach the diagnosis of May Thurner Syndrome. This diagnosis is based on a set of clinical, hemodynamic and morphological criteria.
- When May Thurner Syndrome is diagnosed, stenting supported by angioplasty is simple, minimally invasive, and obtains excellent results both in terms of eliminating symptoms and improving quality of life for the patient.
